# Chilean Consumer Law and Policy: A Brief Outlook

**DOI:** 10.1007/s10603-022-09505-8

**Published:** 2022-01-26

**Authors:** V. Andrade

**Affiliations:** Santiago, Chile

**Keywords:** Chile, Consumer law, Chilean Consumer Protection Policy, SERNAC

## Abstract

Chilean consumer law and policy has a short but enthralling history. As compared with other jurisdictions such as EU countries, its bedrock is still in formation, particularly pertaining to its envisaged role in the regulatory spectrum applicable to consumer markets. However, this circumstance has not been a serious obstacle to several reforms carried out in recent years aimed at broadening its scope of action, including rethinking a proposal regarding the policy goals to be addressed by the national consumer agency, SERNAC. In that context, this article presents a brief account of the main features of Chilean consumer law, considering the current socioeconomic development level of the country, the main pieces of legislation concerning consumer protection in the Chilean legal framework—notably the Chilean Consumer Protection Act—as well as key elements of the public policy approach in this matter. A few examples of topics such as product liability and sale and purchase of consumer goods are also analysed in order to allow a comparison of the Chilean experience in consumer protection with other jurisdictions.

## Socioeconomic and Political Context

Chile is a country located in the so–called Southern Cone of South America, bordering on Perú to the north, on Bolivia to the northeast, and on Argentina to the east, with its western geographic boundary being the Pacific Ocean.

Chile’s form of government is a presidential republic, with the duties of head of state and head of government performed jointly by a president elected by universal suffrage. Both the President of the Republic and the National Congress are involved in drafting national laws, reason for which they are jointly referred to as co–legislators. The National Congress is a bicameral legislative body, consisting of the Chamber of Deputies and the Senate.

Although in administrative terms Chile is divided into “regions” (currently 16), its structure is that of a unitary state. In the area of trade and international commerce, Chile is a signatory party to some 30 international free trade agreements and international cooperation treaties, notably the Association Agreement with the European Union in 2002 (in force from February 2003), the Free trade Agreement with the United States of America of 2004, and recently the Comprehensive and Progressive Agreement for Trans–Pacific Partnership (CPTPP or TPP–11) whose ratification by the Chilean Senate is still pending (agreement establishing an association between the European Community and its Member States, of the one part, and the Republic of Chile, of the other part, 2002, pp. 3–1450; U.S. Chile Free Trade Agreement, 2004; The Comprehensive and Progressive Agreement for Trans–Pacific Partnership, 2018).

Due to its past as a colony of the Spanish crown (1541–1818), like most of the countries of South America Chile is usually considered a country of Western culture. Its legal system fits into the civil–law tradition, in which the main source of law is the statute (positive law), with the role of courts essentially being limited to judging specific cases. The rulings of the superior courts (“case law”) do not constitute a mandatory legal precedent for lower courts, except in very specific cases. Notwithstanding, they are still a relevant source for judicial construction of the law and legal reasoning (so–called *jurisprudencia*).

Table 1 summarizes some of the more pertinent indicators of the economic and social development of Chile.

**Table 1:** Main indicators for Chile

Indicator	Value
Population (2019)	18 952 035
Total surface	756 096 km^2^
GDP (2019)	279 385 MM USD
GDP *per capita* (2020, at current 2021 USD price)	13 231 USD
Household consumer expenses (2020 % of GDP)	59.063
Life expectancy at birth, total (years)	80.81

Source: DataBank—World Bank (2021)

## Features of Chilean Consumer Law

In Latin America, it was only in the late 1980s and early 1990s that countries in this region began to adopt omnibus laws on consumer protection, distinguishable from other types of regulation existing until then in that, among other matters, they referred to the conditions of supply of specific essential goods, monitoring of authorized weights and measures, and prohibition of fraudulent or deceptive behaviour in the marketplace. This “tardiness” in adopting legal measures that would take particular care of consumers’ interests in Latin America, compared to the situation in the USA and many European countries, can be explained by a combination of various factors. On the one hand, prior to industrial privatization in the 1980s and 1990s, a significant part of public utilities and services and primary goods were either provided by the state or distribution/delivery was controlled by the state, which, within this role, did not tend to regulate the conditions applicable to the relationship between consumers and suppliers of these products and services but, rather, implemented other public policy goals, in areas such as improving public health, boosting connectivity, and increasing the availability of certain consumer goods (Engel, Muñoz & Repetto, 2013, pp. 5–6). On the other hand, only in the early 1990s, the level of development of Latin American economies was stable enough for the appearance of interest groups and social movements, though with different levels of sophistication, demanding better treatment for consumers in the market. In the same way, as a result of initiatives by various international movements and gradual identification by a growing middle class of the harmful effects of the so–called neo–liberal economy taken together with globalization, governments gradually began to take consumers’ interests into consideration in their legislative agendas (Baker, 2009, p. 265; Rhodes, 2006).

Considering this context, since 1997, Chile has a consumer protection act in the shape of Law No. 19,496 (the Chilean Consumer Protection Act or CCPA, 1997) as a result of a lengthy legislative process of over six years in the National Congress (Engel, 1998, p. 21; Manzano, 2006, p. 6). Since its entry into force in 1997, the CCPA has been amended at least 16 times. One of the latest of those amendments, which will be dealt with in greater detail below, consists of a reform which was originally aimed at overhauling the institutional framework of the Chilean consumer agency, the National Consumer Service (*Servicio Nacional del Consumidor*, SERNAC) by means of granting it greater powers, including authorization to issue regulatory standards and impose sanctions (a power originally conferred solely on courts), along with improvements to compliance and enforcement mechanisms in consumer legislation by redesigning the system of fines applicable to suppliers in cases of breach of CCPA provisions.

To summarize the extent of the original text of the CCPA, it can be observed that it considered matters such as the following: (1) legal definitions of “consumers” and “suppliers” and provisions pertaining to the substantive scope of the law; (2) a regime and by–laws for intermediary bodies representing consumer interests (designated as “Consumer Associations”); (3) regulation of contracts of adhesion, general contractual terms, and unfair terms, based on the regulatory technique of a “black list” of terms, plus a general clause on unfairness subsequently added; (4) suppliers’ liability for defective products and negligence in provision of services; (5) provisions on consumer safety; (6) mandatory disclosure duties, mainly in the form of the umbrella concept of “Basic Commercial Information”, which is defined without any changes in Article 1(3 N°1) CCPA as “the data, instructions, documents, or indications that the supplier is required to provide to the public in compliance with a legal standard”; (7) a framework for advertising, debt collection, and other commercial practices; (8) mandatory terms in consumer credit; (9) special proceedings for individual consumer disputes (including damages claims) before municipal courts (*Juzgados de Policía Local*), and years later—in one of its first large–scale amendments (2004)—a collective redress scheme concerning collective and “diffuse interests” of consumers; and (10) a new framework of powers for SERNAC, which was incorporated as a public agency, as legal successor to the former *Dirección de Industria y Comercio*, seven years before enactment of the CCPA, by virtue of Law No. 18 959 of 1990.

Due to the existence of this piece of legislation on the subject of consumer protection, theoretical analysis in Chile has been mainly focused on studying the scope of the CCPA, and its *lex generalis* or *lex specialis* nature, as well as the consequences of its application to contracts applied by and between consumers and suppliers and, later on, how this law should be appropriately construed for B2B relationships, considering that, since 2010, micro and small enterprises are entitled to invoke some of the CCPA provisions when dealing with their suppliers. As to this latter topic, Law 20 416 (known as the “Small and Medium Enterprises Statute” or “SME Statute”, 2010) affirms that regarding micro and small enterprises—defined according to specific turnover thresholds—certain CCPA provisions will be applicable to acts and contracts concluded by them and their suppliers. The SME Statute also clarifies that applicability of the CCPA to micro and small enterprises is aimed at protecting such companies when they act “in their role as consumers”, an expression that has triggered some controversy on the actual scope of the CCPA in B2B contracts executed by micro and small enterprises as a result of their main business purpose. Overall, issues such as those highlighted above have certainly meant that academia and the courts have put more emphasis, on the one hand, on the private law aspects of the CCPA regime, especially when they may diverge from the standards and principles set forth in the codified civil and commercial law and, on the other hand, on the compatibility of its provisions, mainly those related to the mandatory content of contracts, with regulation of regulated markets—comprising both laws and administrative rules issued by supervisory agencies—such as telecommunications, health care services, and financial services.

Without prejudice to the above, with the latest reforms of the CCPA, which are a substantial departure from the liberal and light–touch regulatory approach used in the original wording of the CCPA, the presence and importance of tools of public law in Chilean consumer law show a marked increase, to such an extent that perfecting and developing such tools forms a key part of the most recent proposals in the area of consumer protection policy (Yeung, 2010, p. 64).

Thus, what began as a “framework” act on consumer protection, without detailed regulation of any specific economic activity—except for the sale and purchase of goods (both perishable and durable goods), together with a very limited set of provisions concerning the liability of service providers (Articles 25, 40, 41, 42, and 43 CCPA, 1997)—has led to a comprehensive piece of legislation, intended to apply to as many economic activities as possible, notably by means of a default law provision in the case of regulated industries (Article 2 CCPA, 1997), mixed with quite detailed regulation for certain products and services, ranging from consumer credit to motor vehicle parking services (Article 49 CCPA, 1997), even including a special mandatory disclosure rule applicable to video games sales (Article 15–A, 15–B, and 15–C CCPA, 1997).

**Fig. 1:**
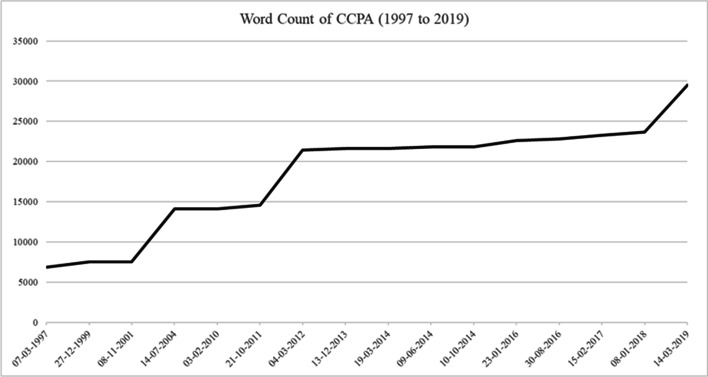
Evolution of the total word count of the CCPA through 1997–2019. Source: prepared by the author based on information retrieved from the Chilean National Congress Library (www.bcn.cl)

An overview of the amendments introduced to the CCPA since it has been in force is presented in Table 2. In turn, Fig. 1 shows how the extent of the CCPA has evolved through its reforms over the 20 years since its promulgation, measured in terms of the total number of words of the legal text, starting from the original 6,886 to the current 29 558. This implies that the law, measured in this sense, has quadrupled in extent, as can be confirmed by the three most extensive reforms that it has undergone (in 2004, 2011, and 2018–2019). Finally, Table 3 shows some pieces of legislation other than the CCPA in which rules belonging to consumer law can be observed.

**Table 2:** Amendments to the CCPA (1999*–*2021)

Amending law	Date (entry into force)	Topic
19 659	December 27, 1999	Debt collection
19 761	November 8, 2001	Debt collection and consumer credit
19 955	July 14, 2004	Collective redress, CCPA pre–emption and the *lex specialis* principle, distance and electronic contracts, misleading information, SPAM, powers of SERNAC, consumer case law registrar
20 416	February 3, 2010	Micro and small enterprises as consumers
20 543	October 21, 2011	Enhancing collective redress
20 555	March 4, 2012	Consumer credit and financial services, financial arbitrators and mediators, SERNAC certifying officers
20 715	December 13, 2013	Consumer credit, debt collection, and interest rate ceilings
20 720	March 19, 2014	Insolvency nomenclature
20 756	June 9, 2014	Labelling of video games
20 855	January 23, 2016	Mortgage cancellation
20 945	August 30, 2016	Collective redress in antitrust proceedings
20 967	February 15, 2017	Parking lots (fees and liability of parking operators)
21 062	January 8, 2018	Debt collection
21 081	March 14, 2019 (partially) to September 14, 2020	Strengthening of SERNAC, new out–of–court collective proceedings, increase of fines, new mitigating and aggravating factors for suppliers’ liability
21 236	September 8, 2020	Portability of financial products and services (right–to) and financial information
21 320		Debt collection—calls and other debt collection activities during COVID–19 pandemic

**Table 3:** Other statutes concerning consumer law topics in the Chilean legal framework

Law N°	Date (original publication in the Official Gazette)	Main consumer law topic
18 010	June 27, 1981	Loans and other credit operations—including consumer credit—interest rates, and interest rate ceilings (“usury laws”)
Decree with Force of Law 1/2005 in re. Decree–Law No. 211	March 7, 2005	Competition Act
18 168	October 2, 1982	General Telecommunication Act (rights of telecommunication service users and net neutrality)
19 628	August 28, 1999	Data Protection Act
20 009	April 1, 2005	Payment card fraud (liability for)
20 453	February 3, 2010	SME statute (small companies as consumers)
20 584	April 24, 2012	Patients’ rights
20 606	July 6, 2012	Nutritional facts and food labelling
20 667	May 9, 2013	(Chilean Commercial Code amendment) Insurance contracts (including collective insurance policies)
20 606	July 6, 2012	Nutritional facts and food labelling
20 831	April 30, 2015	(Aeronautical Code amendment) Air passenger rights
21 236	June 9, 2020	Financial services and products portability (right to)

### The Latest Comprehensive Amendment to the CCPA: The Physiognomy of SERNAC

The administration of Michelle Bachelet, in her second term as president (2014–2018), was led for several reasons to propose a redesign of the institutional scheme of SERNAC as a consumer agency, within the framework of structural reforms in terms of regulation of the economy. These factors consisted of a combination of high–profile cases in sensitive sectors for the citizenry, including the financial sector and public utilities, and a noticeable increase in the use by SERNAC of enforcement and advocacy tools not expressly conferred by law, including the development of so–called collective mediation, publication of soft–law position papers, and use of its powers to request information as a mechanism for demanding changes in suppliers’ behaviour.

Collective mediation was the name given to out–of–court negotiations carried out before the start of any legal action in court by SERNAC that the agency conducts with suppliers regarding an alleged breach of the CCPA which affects collective or diffuse interests of consumers. The term “collective mediation” itself is an improper designation for this type of negotiation, since SERNAC does not act as a third party mediator between consumers and suppliers, but rather as a party interested in reaching a solution for a circumstance that according to prior evaluation performed by SERNAC would qualify as a breach of the Consumer Protection Law that affects collective or diffuse interests. As a general rule, a successful collective mediation ended with a non–self–enforceable commitment by the supplier in order to cease the alleged conduct, compensate the affected consumers, and perform an audit on the level of compliance of its commitments to SERNAC.

The use by SERNAC of powers to require information as a mechanism for demanding changes in suppliers’ behaviour was critically reviewed by the Comptroller General of the Republic in its Opinion No. 71,055 of 2013, which states that “[I]t should be highlighted that this service [SERNAC] does not have the authority to give orders to banking institutions about the measures they must adopt regarding issues with their clients*.*” An often highlighted element of this policy was a critical assessment of the performance of SERNAC at that time, which would justify the need to improve its design: SERNAC was depicted as a “toothless lion” (Biblioteca del Congreso Nacional, 2018, p. 50) since although it is claimed by law to protect consumers from potential abuses by suppliers, it did not possess suitable tools to prosecute and apply sanctions that could adequately deter suppliers from engaging in behaviour infringing the CCPA, bearing in mind that SERNAC, strictly speaking, had filing of judicial proceedings before the courts as its main tool for enforcement. According to what was then Article 58 (f) CCPA, SERNAC has the power to handle consumer claims and then report on them to suppliers in order that the latter could voluntarily propose a solution. Based on that authority, SERNAC has developed a free–of–charge platform throughout the nation to handle claims, responses, and notification for consumers and suppliers (titled “SERNAC Facilita”). For some authors, the platform should be treated as an administrative enforcement tool (Momberg et al., 2018, p. 155).

A significant element for underpinning this assessment was a comparison of SERNAC’s institutional framework, in terms of resources, structure, and powers, with other supervisory or oversight agencies, namely the National Economic Prosecutor Office (*Fiscalía Nacional Económica*—FNE) and the sectoral agencies, which in Chile often adopt the denomination of “superintendencies”.^1^ These possess explicit powers conferred by law to prosecute, investigate, and (in the case of most superintendencies) impose sanctions within their specific regulatory perimeter (Biblioteca del Congreso Nacional, 2018, p. 4).

Thus, in June 2014, the President of the Republic filed a bill for legal reform with Congress aiming to strengthen SERNAC together with other relevant changes to the CCPA, including adoption of a system of administrative dispute resolution as an alternative to the courts, explicit regulation of collective mediations before SERNAC as “voluntary collective interest proceedings”, increases in fines, improvement of the legal regime for consumer associations, and an increase in the period of the statute of limitations for CCPA–based claims.

After more than three years of intense debate in the legislature, the proposed law, with modifications from its original text, was approved by both chambers of Congress and submitted in October 2017 to the Constitutional Court of Chile (CCC) for a regular *ex–ante* constitutionality review of the law.

In a both unforeseen and controversial ruling for Chilean constitutional law (CCC Ruling No. 4,012–17 (January 18, 2018) regarding “Bill of Amendments to Law No. 19,496 (Bulletin No. 9,369–03), particularly due to the significant *ex officio* exercise of its powers by the CCC in the review of the bill regarding provisions on which it was not expressly requested to issue its opinion, the CCC declared the following sections of the draft law as unconstitutional, rendering their publication as national law not feasible: The new sanctioning procedure before SERNAC (which was understood as “jurisdictional” and therefore, under Article 76 of the Chilean Constitution, assigned only to courts of law), the new rule–making powers of SERNAC are a threat to the principle of legal domain, and the enhanced power to require information conferred on SERNAC includes information subject to secrecy or confidentiality restrictions by other public agencies.

On this subject, regarding sanctioning powers, the point highlighted by the CCC in Recital 33 of its ruling is particularly important:

33rd: That, in violation of Articles 19, paragraph no. 3, sixth paragraph, and 76, first paragraph, of the Constitution, firstly, the rules of the Draft Law that replace the current regime of separation of powers with a new one, *which unifies administrative and jurisdictional powers*, are declared unconstitutional.

This is because while under the current regime, the National Consumer Service performs some oversight duties that belong to the state administration, while leaving in the hands of the competent courts the sanctioning and correction of infringements of the respective regulations, in the draft law, said public service would assume jurisdictional powers to act as an arbiter to resolve disputes, impose sanctions on suppliers, and adopt all sorts of preventive and precautionary measures, in circumstances in which such measures can only be adopted by an independent and impartial tribunal, characteristics which it does not possess.

This ruling, then, does not call into question the regulations that assign such powers for consumer protection to the courts; *the objection is that they cannot be held by a solely administrative body, in virtue of a basic principle of universal public law, that is, the separation of powers *[…] (emphasis added)

Thus, following the CCC ruling, on September 13, 2018, Law No. 21,081 was finally published in the Official Gazette, except for the sections that were declared unconstitutional. Despite the changes undergone due to its review of constitutionality, Law No. 21,081, which entered into force in March 2019, in any case entails a series of major reforms to Chilean consumer legislation, including the following: (1) the oversight powers of SERNAC are strengthened, including the possibility of entering suppliers’ premises to conduct evidentiary diligences; also, it is granted interpretive powers regarding the CCPA and other consumer law–related statutes, albeit limited to harmonizing criteria concerning suppliers within that agency and therefore not binding on suppliers; (2) the public budget for SERNAC is increased and its internal structure is modified; (3) the regime applicable to consumers associations is modified, increasing the flexibility of regulations on their financing and their range of action; (4) “voluntary collective interest proceedings” before SERNAC, as a non–adversarial procedure—aimed to succeed the above–described informal collective mediations—for the protection of collective interests, with *erga omnes* effect when the final resolution is approved by the Court;^2^ (5) modifications of judicial proceedings are introduced, including rules on the inadmissibility of appeals in individual proceedings involving small claims. Additionally, the former restriction on compensation for non–pecuniary damages in collective proceedings is derogated from the CCPA; (6) fines for infringements of the law are increased, and a regime of mitigating and aggravating factors for liability is included; (7) a regulation on automatic compensation in the case of suspension of provision of services is included in the CCPA as a resemblance of similar provisions in the General Telecommunication Act; (8) the statute of limitations for breaches of the CCPA is extended from the original six months to two years after infringing conduct ceases.

### Chilean Consumer Protection Policy

State policy in consumer matters is mainly exercised through the powers performed by SERNAC.

Under Article 57 CCPA, SERNAC is a public service, decentralized in functional and territorial terms, with its own legal personality and budget, subject to oversight by the President of the Republic through the Ministry of the Economy, Development, and Tourism.

The geographically decentralized nature of SERNAC is reflected in the existence of regional directorates in each region of Chile, which are subordinate to the National Directorate of SERNAC. In the Chilean institutional framework, SERNAC plays the role of a consumer protection agency and, as such, participates as a representative of the Chilean state in various international cooperation forums and organizations, such as the OECD Consumer Policy Committee, the Forum of Ibero–American Government Consumer Protection Agencies (FIAGC), and the International Consumer Protection Enforcement Network (ICPEN).^3^

SERNAC forms an integral part of the state administration, conceived as being at the service of the people; therefore, its essential purpose is to “promote the common good by taking care of public needs on a continuous and permanent basis and stimulating the country’s development” (Article 3 Law No. 18 575 of General Bases for the Administration of the State).

The role of promoting the common good that is formally exercised by SERNAC in the area of consumer protection has been specified by the comptroller entity for the public administration, namely the Comptroller General of the Republic, through its Opinion No. 23 404/2010, highlighting the following on this subject:

Defence of consumers is a goal that the State has adopted and that is implemented through the action of the SERNAC: (…) It appears from the provisions mentioned that the National Consumer Service constitutes a public service created by law in order to safeguard the interests of consumers and that it is empowered to engage in legal action in specific circumstances. From the above, *it can be inferred that defending the interest of consumers is a specific goal of the stat*e that is implemented by means of the National Consumer Service, which may, for said purposes, appear in court (…) (8° y 9°) (emphasis added) (CGR Opinion No. 23 404/2010)

Since the amendment introduced to the CCPA by Law 21 081, SERNAC is designated at the same time as an oversight agency belonging to the state administration, as is the case of superintendencies and other regulatory bodies.

As established by paragraph 1° of Article 58 CCPA, the fundamental mission of SERNAC is “to oversee compliance with the provisions of the present law [the CCPA] and other regulations that govern relations with the consumer, to publicize the rights and duties of the consumer and perform actions to inform and educate consumers”. In order to enact its stated mission, the legal framework grants SERNAC a series of specific duties associated with various public powers for this purpose, which are mainly set out in Article 58 CCPA.

To mention the most relevant, these include oversight of compliance with the CCPA and “all other regulations concerning consumers’ rights”; administrative interpretation of consumer regulations, which will only be binding on its officers; similar to the National Economic Prosecutor’s Office, to propose all amendments to or derogations from rules necessary for adequate protection of consumers’ rights; to provide information and to respond to consultation by public bodies involved in protection of consumer rights; to initiate voluntary collective interest proceedings; to develop and put in place educational and informational programmes; to request studies of products sold in the marketplace; to gather information from research and studies on consumer issues; to oversee compliance with legal and regulatory provisions related to consumer protection by filing legal actions with the judicial and administrative authorities; and to certify financial contracts as to their compliance with the standards contained in the CCPA and its regulations.

Although to date no official document exists establishing general guidelines to be followed by SERNAC in the performance of its duties, at least four features can be distinguished that have characterized its approach to consumer issues in recent years.

First is a tendency to perform quasi–regulatory powers through instruments of *soft law* (Tsagas, 2016, p. 105). As pointed out previously, in the absence of expressly granted rule–making powers, SERNAC has begun to publish a series of “Guidelines on Legal Issues”^4^ that contain the position of the agency on various legal subjects and the legal basis for this, with the goal that suppliers should know which interpretation will guide SERNAC when legal action is brought before the courts. Currently, since the 2018 reform, these quasi–regulatory powers are exercised by virtue of the issuance of administrative interpretations of law which, as mentioned, are only binding for its officials.

In any case, despite the limitations on their legally binding nature, the “Interpretative Circulars” (as they are nowadays referred to) embody the institutional position of SERNAC on the CCPA and other consumer–related laws, as well as the expectations of the agency regarding business conduct of suppliers, ranging from topics such as financial products and consumer claims handling to COVID–19–related measures and its impacts on contract performance (Id.).

The second feature is a significant increase in the use of collective mediation, in contrast to the use of legal actions on behalf of collective or diffuse interests, as a mechanism to obtain binding commitments from suppliers and financial compensation for consumers. This practice, as indicated above, was finally formally established in the CCPA on the occasion of its latest reform, through a special section on “Voluntary Collective Interest Proceedings”, which regulates their conditions, timeframes, and results.

Third is the increased concern as to the consequences for consumers of infringements of antitrust law, especially in the case of collusion and other unlawful activities by businesses. This has been represented by both (1) the pursuit of consumer representative actions in cases in which the existence of anti–competitive acts has been asserted by the Chilean Competition Tribunal which nowadays—by virtue of Law No. 20, 945—includes the possibility to bring CCPA collective redress proceedings before said court and (2) a higher level of coordination with the agency in charge of prosecuting infringements of antitrust law and regulations, the FNE (OECD, 2014, p. 104).

Some decisions of the Chilean Competition Tribunal include *ODECU v. Farmacias Ahumada*, Docket No.10 351–2013, 25° Civil Court of Santiago; *SERNAC v. Agrosuper et al*, Docket No. 28 470.2015, 29° Civil Court of Santiago; *SERNAC v. Pullman et al*, Docket No. 22 416–2015, 13° Civil Court of Santiago; and *SERNAC v. SCA Chile S.A*., Docket No. 1374–2016, Municipal Court of Colina. It must be highlighted that in the last case, related to collusion between SCA Chile S.A. (SCA) and CMPC Tissue S.A. (CMPC) in the “tissue paper market”, CMPC Tissue filed a leniency motion before the Chilean Competition Tribunal. As a consequence, SERNAC initiated a voluntary collective mediation with both companies involved, but only CMPC consented to be part of the proceedings, so that SCA was consequently sued by SERNAC. The collective mediation with CMPC ended with a compensation agreement dubbed the “Tissue Paper Compensation Programme” which includes 7,000 Chilean Pesos (10 USD app.) monetary compensation for each Chilean above 18 years old, accruing to a total of USD 150 000 000.

The fourth feature concerns lack of contact and coordination between SERNAC and the other sectoral supervising agencies. In contrast to what would seem to occur regarding the FNE, in recent years, lack of communication or, more accurately, of coordination between agencies overseeing the functioning of highly regulated markets (e.g., telecommunications, insurance, banking, electricity services, health care authorities) and SERNAC has become evident. For part of legal academia, this problem could be resolved by strengthening the mechanisms and instances of cooperation between the public agencies concerned, bringing to SERNAC the same powers and resources currently conferred on agencies such as superintendencies, and then redistributing the authority to oversight of technical and prudential requirements (keeping them in the hands of sectoral agencies) and, consequently, assigning to SERNAC rulemaking and enforcement duties related to market conduct (Pardow, 2015); all of this is in a way similar to the financial supervision model known as “twin peaks” (Haentjens, & Gioia-Carabellese, 2015, p. 97).

### Consumer Law Generations in the Chilean Legal Framework

In view of the gradual modernization of consumer protection standards, mainly through stepped reforms of the provisions of the CCPA and approval of specific bodies of legislation with rules envisaged to protect users and consumers, it becomes difficult to assign the Chilean consumer protection system to a specific generation.

However, it can be acknowledged that in principle, and due to the considerations observed for its enactment, the CCPA, as originally passed, constitutes an emblematic example of the first generation of consumer regulation. Thus, by closely following the model for basic rights stated in the “Consumer Bill of Rights” to which US President John F. Kennedy referred in his speech in 1962 as the list of consumers’ “legitimate needs” (Kennedy, 1962) also enshrined in the United Nations Consumer Protection Guidelines of 1985, the CCPA was developed under the premise that consumers’ interests deserve special legal treatment, consequently granting consumers a series of basic rights to be exercised in regard to the various providers of goods and services on the market (Article 3 CCPA, 1997).

Nonetheless, some regulatory developments can be attributed to the so–called second generation of consumer law, since special regimes are established governing certain products and services, with respect to both substantive and procedural regulations.

One of these is the set of regulations that protect consumers of financial products and services. With the entry into force of Law 20 555 (“the Financial SERNAC Act”) in 2012, Chile joined a worldwide trend of including protection of consumers of financial products and services as a key part of public policy on financial markets, beyond prudential regulation aimed at reducing the risks to which financial institutions and their depositors’ money are exposed. This act constitutes a prominent example of the informational paradigm in consumer law since it was conceived upon the understanding that “the best way to protect consumers is improving the available information for their consumption decision making” (Biblioteca del Congreso Nacional, 2011b, p. 5).

Said statute, which was particularly designed for consumer credit products, added to the CCPA, among other things, (1) a specific list of rights of consumers and users of financial products and services, (2) a minimum contractual term for this type of contracts, (3) the duty to provide standard commercial information, including a form summarizing the most relevant contractual terms (“*Hoja Resumen*”) and disclosure of a comparative index of the financial (total) cost of a credit product, the Annual Percentage Rate “*Carga Anual Equivalente*” (Andrade, 2015, p. 51), (4) certification of financial contracts by SERNAC, and (5) the formal existence of an alternative dispute resolution system through each supplier’s customer assistance systems and voluntary membership in a system of financial mediators and arbiters.

Another case in which this tendency appears, once again in the scope of financial services, relates to the new regulation on insurance contracts that was included in the Code of Commerce with the entry into force on December 1, 2013, of Law 20 667. This reformed regulatory framework, which is applicable both to relations between professionals (B2B) and between professionals and consumers (B2C), established a series of provisions protecting insured persons. These are mandatory (under the so–called non–derogability principle), except in the case of (1) property and casualty insurances contracted on an individual basis (that is, not collective insurance), as long as both the insured party and the beneficiary are legal entities and the amount of the agreed annual premium is greater than 200 Unidad de Fomento (UF), and (2) maritime and air hull and transport insurances (Article 542 Chilean Commercial Code, 2013). As can be seen, in this case, the definition of a non–professional protected party (the insured) is given not only by a series of specific features of their position in the contract and in the marketplace (as is the method used by the CCPA to define the consumer), but rather, through of a series of objective elements of the insurance contract, including a monetary “threshold”, which define the mandatory or default nature of substantive regulation.

Finally, there is a notable surge in the use of regulatory tools in line with insights from behavioural economics (Mullainathan & Thaler, 2000; Barr, Mullainathan & Shafir, 2013), such as the use of summary information tools on certain product features, e.g., the *Carga Annual Equivalente* in consumer credit, noted above, and more recently, regulations on food labelling and advertising and the introduction of “Stop Signs” with “high in” warnings concerning the level of calories, sodium, fat, and sugar. Thus, as was indicated in the legislative procedure for this law, “as occurs in most countries in the world, the idea is to guide consumers’ behaviour through clear indications and information on the quality and quantity of the food item being consumed, as well as to encourage healthy behaviour patterns and at the same time, provide warnings of the risk of consuming food items harmful to health, with the aim of contributing to the duty of reducing the risk factors that are predominant in the current era” (Biblioteca del Congreso Nacional, 2011a, p. 4).

## Comparative Law Influences

In its substantive dimension, Chilean consumer law has been influenced by the experiences of several Latin–American and European legal systems. These regularly serve as benchmarks of comparison in terms of the “protectiveness” of the CCPA (for example, statutory guarantee rights, time frame for exercise of the right of withdrawal, mandatory contract terms, and so on). An important part of the legal framework for control of unfair terms in contracts of adhesion can be traced to the concepts developed in Directive 93/13/EEC, namely, the definition of a restricted list of clauses considered abusive due to their content (a “blacklist”), as well as a final general unfairness clause (Article 16 g CCPA, 1997) similar to Article 3 of Directive 93/13/EEC.

Another example of the clear influence of EU law is regulations on protection of financial consumers added to the CCPA in 2011, where the paradigm of mandatory information disclosure as a tool to protect consumers finds one of its most vivid expressions. Both the emphasis on transparency regarding the intrinsic features of credit products and standardization of their essential contractual features, aspects added to the CCPA by Law No. 20 555, can be observed in Directive 2008/48/EC, including the *Carga Anual Equivalente* (similar to the annual percentage rate of charge) and the *Hoja Resumen* for financial agreements (akin to Standard European Consumer Credit Information).

In other aspects, the definitions of consumer and end user have taken into account certain developments in Argentine and Spanish law (notably, the inclusion of end users and the notions of material and legal consumer). According to Article 1 (1) of the CCPA, a consumer is any “natural or legal persons who, by virtue of any legal act for consideration, acquire, use or enjoy goods or services as end user”. In a more holistic dimension, the constitutional enshrining of consumer protection in the legal systems of Mexico and Brazil has been a source of inspiration for a large number of legal initiatives that aimed to amend the Chilean Constitution by adding consumer protection as a constitutional guarantee.

In terms of collective redress mechanisms, the Brazilian approach on “supra–individual interest” was closely followed by reform which introduced representative actions in the CCPA (Aguirrezabal Grünstein, 2006, p. 83).

In turn, regarding the institutional design of the consumer protection agency, it must be acknowledged that the spectrum of reference is somewhat wider. Thus, the regulatory models based on independent agencies such as the FTC and the CFPB in the USA and the CMA in the UK constitute significant experiences for the understanding and potential improvement of the role of SERNAC, especially in view of the current convergence between the new means of regulatory intervention and accountability and application of the findings of behavioural economics.

Lastly, it cannot be left unmentioned that since Chile joined the OECD in 2010, studies, surveys, and benchmarks conducted by that international cooperation forum end up as a common frame of reference for review of consumer protection policy issues within the Chilean legal framework.

### Scope of the Chile–EU Association Agreement (2002)

As stated earlier, in 2002, Chile and the EC (from 2009, the EU) signed an Association Agreement (the Chile–EU AA, 2002) which aimed to cover “the political, commercial, economic and financial, scientific, technological, social, cultural and cooperation fields,” although “[i]t may be extended to other areas to be agreed upon by the Parties” (Article 2(3) of the Chile–EU AA, 2002).

As to economic cooperation between the signatory parties, the Chile–EU AA includes provisions pertaining to consumer protection as set forth in its Article 29:

Article 29 – Consumer protection

Cooperation in this field shall seek to make the consumer–protection programmes in the Parties compatible, and shall as far as possible cover:

*(a)* making consumer legislation more compatible, so as to avoid trade barriers;

*(b)* establishing and developing mutual information systems for dangerous goods, and interconnecting those systems (early–warning systems);

*(c)* exchanges of information and experts, and encouraging cooperation between both Parties’ consumer bodies; and

*(d)* organising projects for training and technical assistance.

As can be seen, the scope of cooperation between the EU and Chile in consumer law is not focused on enforcement or harmonization of certain rules but on a mutual exchange of public policy approaches on a long–term basis. This differs from the nature of obligations set forth in other agreements between the EU and Latin American countries such as Perú and Colombia, which agreed to put in place effective measures to protect consumers in e–commerce transactions, including cooperation between consumer protection authorities (Article 166 Trade Agreement between The European Union and its Member States, of the one part, and Colombia and Perú, of the other part).

The most concrete evidence of the outcome of committed cooperation has been the use by SERNAC of safety alerts published in the Rapid Alert System for Non–Food Consumer Products (RAPEX) for which the basis was established in the General Product Safety Directive 2001/95/EC (GPSD) as a source of its own safety warnings regarding consumer goods, such as cars, electronic devices, and toys.^5^

## Case Studies on Product Liability and Consumer Products

### Product Liability and Product Safety

Under Article 3(d) CCPA, consumers have the right to safety in consumption of goods and services, including protection of their health and the environment. A curious feature of the CCPA is that when the act enunciates the rights recognized for consumers, at the same time, certain “consumers’ duties” are explicitly referred to (in fact, the corresponding preamble of paragraph 1° of Title II CCPA 1997 is Rights and Duties of Consumers). As such, in the case of safety, consumers have the duty to avoid risks that may affect them.

Safety standards are highly dependent on the nature of the product or service concerned. However, the CCPA includes a general duty to provide consumers with operation manuals or other user instructions, as part of the “Basic Commercial Information” when regular use of a good or services implies a certain risk for the integrity or safety of individuals (Paragraph 3 Article 3 CCPA, 1997). Regarding the content of said documentation, the law states that this must be comprehensible, readable, and written in Spanish (Article 32 CCPA, 1997).

Although safety standards (and labelling requirements) are issued by different authorities within their respective regulatory perimeter—notably the Superintendence of Electricity and Fuels, the Ministry of Health, the Undersecretary of Transportation, and the Chilean Financial Market Commission—SERNAC, as part of its educational goals, informs consumers about safety standards and labelling requirements in a section of its institutional website.^6^ Additionally, during recent years, SERNAC has filed judicial actions against suppliers which do not properly follow sectoral safety standards as a case of breach of the safety right granted in the CCPA. This is besides administrative proceedings conducted by sectoral authorities against regulated suppliers when they do not meet regulatory requirements on disclosure of information, third–party certification (when applicable), and labelling.

Furthermore, goods and services qualified as “dangerous” are subject to stricter rules (Articles 44, 45, 46, 47, 48, and 49 CCPA, 1997) pertaining to precautionary measures to be implemented by suppliers, including mandatory notification to the competent authority of any previously unforeseen dangers and risks.

When a Court or pertinent authority declares the toxicity or dangerousness of a product, the manufacturer, importer, and first distributor or service provider will be held jointly and severally liable for any harm to a consumer.

Regarding consumer redress, the CCPA grants the consumer a judicial action for damages when a supplier acting negligently in selling a good or rendering a service causes harm to a consumer due to failures or deficiencies in the quality, quantity, substance, origin, safety, weight, or measurement of the corresponding good or service (Article 23 CCPA, 1997).

Finally, it should be highlighted that when “safety” is an advertised feature of a certain product (e.g., non–toxic pencil or ozone–free emissions), regulation on misleading advertising enters on the scene, since it is deemed a breach of the law when a supplier, by means of advertising, knew or should have known leads to error or deception in the consumer regarding features such as non–contaminant, non–risky to health or being recyclable or reusable (Article 28(e) CCPA, 1997).

### Consumer Products

It is commonly said that CCPA remedies on consumer products are based on lack of a conformity standard rather than a regular breach of sale contracts referred to in the Chilean Civil Code rules (Barrientos Camus, 2013, p. 77; Ferrante, 2018). Accordingly, remedies applicable by virtue of the statutory guarantee, the so–called *garantía legal*, are triggered by certain non–conformity circumstances, such as difference in the stated quantity, non–fulfilment of safety or quality standards, defects in manufacturing, hidden or nonapparent defects, differences between the advertised features or description, and the actual delivered good (Articles 19 and 20 CCPA, 1997).

When non–conformity is related to quantity (Article 19 CCPA, 1997), the consumer has the right to request a replacement of the product or a partial refund or credit equal to the excess amount paid. Conversely, when non–conformity is related to other circumstances as noted above, the consumer, within a time–frame of three months (seven days in the case of perishable goods) as from the delivery date of the product, is entitled to exercise what was publicly dubbed as the “triple option right” (Nasser, 2013, p. 541): replacement, repair, or total refund of money (a lay expression for revocation of the contract). In contrast to the rules set forth in Directive 1999/44/EC, the remedies comprised in the CCPA are not based on a “two–tier” system (Howells et al., 2017, p. 35), namely “replacement and repair” as first options followed by termination of the contract as a last resort (or as a default rule).

As a general rule, a consumer must exercise their statutory rights towards the seller of the product. However, in certain circumstances, other entities within the supply chain are liable to comply with remedies for lack of conformity provided by law (Article 21 CCPA, 1997).

Firstly, a consumer who opts to repair a good can exercise their right respectively before either the seller, the importer, or the manufacturer. When non–conformity causes harm to the consumer, the seller, the manufacturer, and or the importer are jointly and severally liable *vis*–*à*–*vis* the consumer.

Additionally, the manufacturer and importer are still responsible for replacement of goods and other statutory remedies (e.g., reduction of price, repair or replacement) when the seller was subject to insolvency proceedings or is out of business by virtue of other reasons.

Finally, the CCPA explicitly states that sellers are entitled to request relief or compensation from the importer or manufacturer when, due to an attributable defect in the goods, said sellers have proceeded to replace or refund money to consumers.

**Fig. 2:**
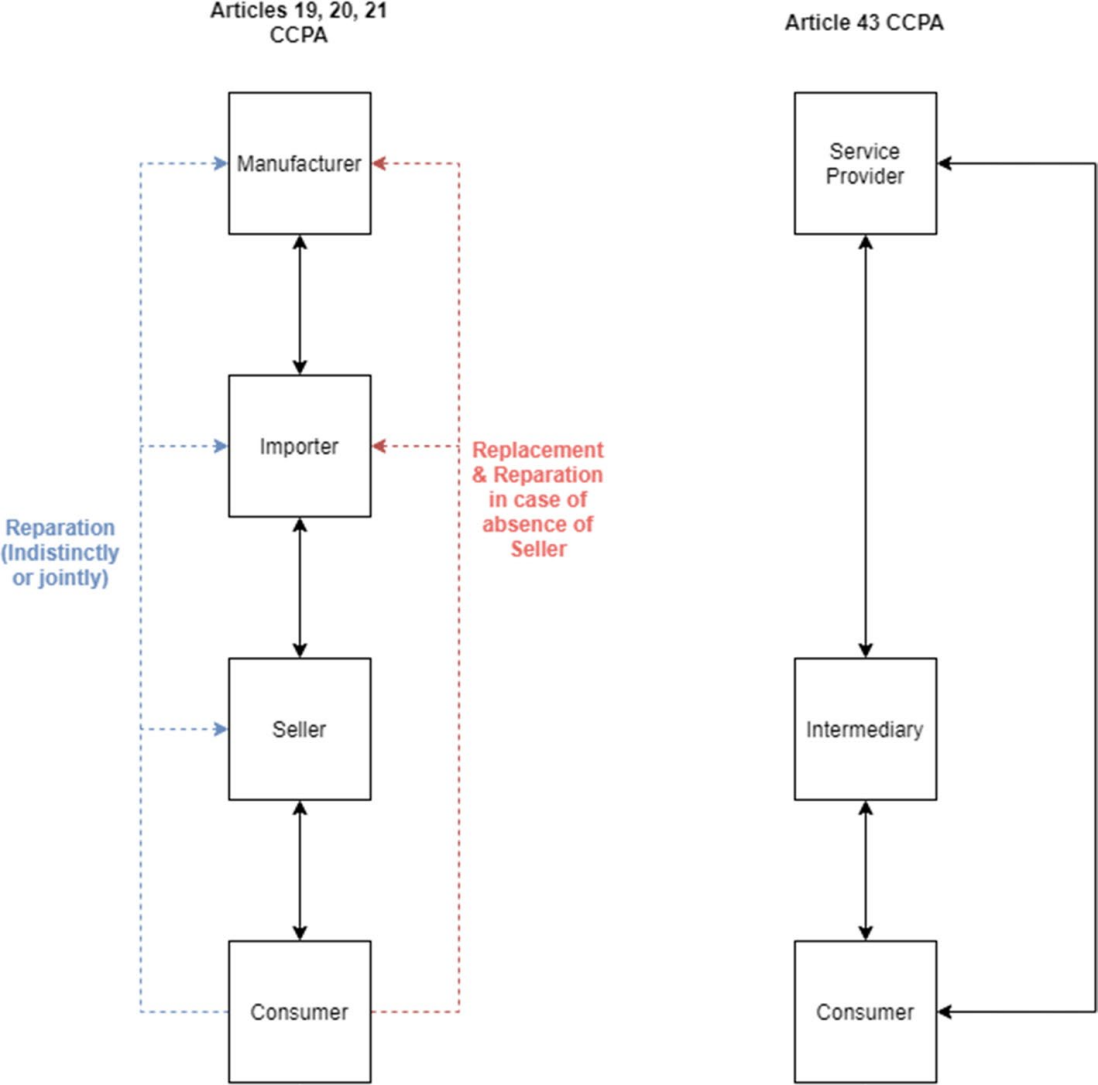
A comparison between statutory guarantee on consumer products (Articles 19, 20, 21 CCPA, 1997) and liability rules regarding service providers (Article 43 CCPA 1997). Source: author

A graphic summary of these rules is presented in Fig. 2, compared with the provisions applicable to services, in which the consumer is entitled to exercise their rights either before the intermediary/broker or the actual service provider (Article 43 CCPA, 1997).

Aside from the non–derogable statutory warranty, the CCPA refers to the case of products covered by guarantees provided either by sellers (conventional guarantees) or manufacturers (extended or manufacturer’s guarantees). When no defect is alleged during the term of a statutory warranty, extended guarantees do not represent a complex legal issue: The consumer must follow the procedures and terms set forth in the guarantee’s terms and conditions to request repair or replacement and, in rare cases, revocation of the contract and consequently a total refund of the price. Conversely, a problem arises when both statutory warranty and conventional guarantees are overlapping. When a product is covered by a conventional guarantee, the consumer must exercise the guarantee *vis–à–vis* the warrantor/guarantor and then exhaust the remedies offered, according to the respective terms of the policy; and only after that, if the defect persists, can it claim the statutory guarantee (Article 21 paragraph 9 CCPA, 1997). This provision has been a source of a huge amount of litigation since there is no clear guideline about what “exhausting remedies” actually means (Soto & Marlene, 2014, p. 583). In recent years, courts have had an understanding, on a pro–consumer interpretation of the law, in favour of the statutory warranty and its non–derogative nature, in which a consumer who exercises the conventional guarantee just once and the defect persists is then fully entitled to the triple option right.

In that regard, SERNAC has brought both collective mediation proceedings and representative actions under the unfair terms provisions of the CCPA, when suppliers by means of the terms of conventional guarantees either (1) deceive consumers about their rights granted by statutory warranty provisions or (2) when they unlawfully restrict such legally granted rights on an *ex–ante* basis.

Finally, it must be highlighted that the abovementioned legal loopholes are intended to be overcome by passing an amendment to the CCPA, which is near to becoming law, and which, among other things, clearly defines the scope of voluntary and statutory protections for consumer products and, furthermore, extends the term of the statutory warranty from three to six months for durable goods (Cámara de Diputados, Law Bill Bulletin No. 12 409–03).

## Concluding Remarks: the Road Ahead

In this chapter, I have sought to offer a summary of the features, roots, and the current state of development Chilean consumer law. Predictably, any assessment of the effectiveness of consumer protection policy in Chile will remain focused for a while on the enforcement mechanisms provided in the CCPA and related regulations. This seems not to be a rare phenomenon taking into account that one of the latest amendments to the CCPA concerning, among other things, the powers granted by law to SERNAC entered into force partially in March 2019 and in full effect in September 2020. However, a change in approach might be necessary in the near future. For instance, there are no substantive standards on cross–border transactions, sharing–economy platforms, and digital content providers in the CCPA, and some remedies designed for distance selling and electronic contracts (whose adoption has increased dramatically due to the COVID 19 pandemic) are poorly implemented, as is the case with the right of withdrawal which can be lawfully contracted out of by the supplier in the respective terms and conditions (Article 3 bis (b) CCPA, 1997). Fortunately, two bills currently under discussion before the Chilean Congress are aimed at properly addressing such pitfalls. One of these relates to regulation of digital platforms, which is in the early phase of discussion (Cámara de Diputados, 2021a, b, Law Bill Bulletin No 14, 561–19). The other, which is just about to become law, deals among other things with establishing at the legal level of a pro–consumer principle of legal interpretation and eliminating the opt–out for the right of withdrawal (Cámara de Diputados, Law Bill Bulletin No 12 409–03).

I am persuaded that substantive measures must form part of the forthcoming legislative agenda. Otherwise, consumers will be left to themselves in a rapidly evolving marketplace.

## Footnotes

1 As of today, there are superintendencies for the following markets and regulated sectors: pension funds, health care insurers and providers, casinos, electricity and fuels, environment, social security, education, water services, and insolvency. As from 2017 and 2019, the former Superintendence of Securities and Insurances and the Superintendency of Banks and Financial Institutions have been jointly succeeded and replaced by the Financial Market Commission (FMC).

2 Article 54 Q CCPA, in force as from March 2019.

3 https://www.sernac.cl/portal/617/w3–propertyname–586.html (accessed 25 November 2021).

4 https://www.sernac.cl/portal/618/w3–propertyvalue–21072.html (accessed 10 October 2018).

5 The safety warnings are compiled by SERNAC and published in a specific section of its institutional website. See https://www.sernac.cl/portal/619/w3–propertyvalue–20990.html (accessed 25  November 2021).

6 Normas de seguridad en Chile - SERNAC: Información de mercados y productos (accessed 26 November 2021).
